# Effect of Silane-Containing Universal Adhesives on the Bonding Strength of Lithium Disilicate

**DOI:** 10.3390/ma14143976

**Published:** 2021-07-16

**Authors:** Yu-Ri Kim, Jae-Hoon Kim, Sung-Ae Son, Jeong-Kil Park

**Affiliations:** 1 Department of Conservative Dentistry, Dental Research Institute, Dental and Life Science Institute, School of Dentistry, Pusan National University, Yangsan 50612, Korea; hn99328@naver.com (Y.-R.K.); song-ae@hanmail.net (S.-A.S.); 2 Department of Dental Education, Dental Research Institute, Dental and Life Science Institute, School of Dentistry, Pusan National University, Yangsan 50612, Korea; jhkimdent@pusan.ac.kr

**Keywords:** lithium disilicate, silane, universal adhesive, microtensile bond strength, field-emission scanning electron microscope

## Abstract

The purpose of this study was to investigate the effect of silane-containing universal adhesives on the bonding strength of lithium disilicate. Two-hundred-and-forty lithium disilicate blocks were divided into 16 groups according to the following surface treatments: hydrofluoric acid (HF)-treated or not, silane-treated or not, and the type of universal adhesive used (All-Bond Universal (ABU); Prime & bond (PB); Clearfil Universal Bond (CU); Single bond Universal (SBU)). After surface treatment, resin discs were bonded to each lithium disilicate using dual-cure resin cement. Bonded specimens were stored in distilled water for 24 h and then subjected to microtensile bond strength (μTBS) test. Failure modes were examined under stereomicroscope. Microscopic observation of bonded interfaces was analyzed using scanning electron microscopy. The μTBS data were statistically analyzed. Regardless of silane treatment, all groups treated with HF showed higher bonding strengths compared to those that were not treated with HF (*p* < 0.05). In groups treated with HF, the bonding strength increased after silane application (*p* < 0.05) except PB and CU (*p* > 0.05). Adhesive failures were dominant in all groups, but some mixed failures were observed in ABU treated with HF and silane. While most of the specimens that were not treated with silane after HF application only showed loose bonding between the ceramic and resin cement due to partial gaps, the specimens treated with silane application after HF showed a tight ceramic–resin interface. In conclusion, the silane in universal adhesives did not effectively improve the bonding strength between lithium disilicate and resin cement.

## 1. Introduction

Glass ceramics are drawing attention as modern dental restoration materials because their coefficient of thermal expansion is similar to that of natural teeth, and they exhibit excellent aesthetic properties, high bending strength, biocompatibility, and low thermal conductivity [1,2,3]. In addition, with the introduction and development of computer-aided design/computer-aided manufacturing (CAD/CAM), glass ceramic restorations, such as veneers, inlays, onlays, and crowns, have become more frequent and uniform manufacturing is becoming possible [4]. Lithium disilicate is a type of monolithic ceramic that has excellent aesthetic properties and superior fracture resistance compared to those of conventional feldspathic porcelains or leucite-reinforced glass ceramics [5].

Excellent materials and a bonding process are necessary for a successful ceramic restoration. The gold standard for ceramic–tooth bonding involves achieving micromechanical retention using hydrofluoric acid (HF) and forming a chemical bond using silane [2,3,6,7,8,9]. HF partially dissolves the glass phase of a ceramic matrix, thereby increasing the contact area and interactions between the luting agent and ceramic [2]. The irregularities on a ceramic surface created by HF etching are reported to be affected by the concentration, application time, temperature, and dilution level of HF [6].

After inducing mechanical interlocking by creating irregularities on the inner surface of a ceramic restoration using HF, a silane coupling agent is applied to bond the inorganic ceramic surface with an organic resin matrix [5]. Silane, which has been used as a bond enhancer since 1977, has two reactive groups: one that reacts with methacrylate and another that reacts with the silica of a hyaline [1]. Silane forms a siloxane bond and increases the surface energy of a ceramic as well as the cement wettability, thereby inducing microscopic interactions between the two materials [3,4].

While all the aforementioned factors are important for the long-term and predictable clinical outcomes of ceramic restorations, there has been demand for materials that can reduce operation time, minimize errors and safety issues during the bonding process, and achieve effective bonding [5].

Universal adhesives are used to bind various direct/indirect restorations with tooth. They have a wide range of applications and reduce operator sensitivity by simplifying the conventional bonding process as “all-in-one” adhesives [1]. 10-Methacryloyloxydecyl dihydrogen phosphate (10-MDP) is one of the major monomers contained in universal adhesives [10]. They have a self-etching ability and are involved in the chemical bonding between hydroxyapatite, metals, and zirconia [3,10].

Some universal adhesives containing silane, which are essential for bonding silica-based ceramics, have been developed. Commercialized universal adhesives include Clearfil Universal Bond (Kuraray Noritake Dental, Tokyo, Japan) and Single Bond Universal (3M ESPE, St Paul, MN, USA). The manufacturers of these adhesives claim that the adhesives reduce the operation process by removing the need for a silane treatment and achieve excellent bonding [5,11].

While the development of silane-containing universal adhesives had a positive effect on the convenience of the operator, reduced chair time, and minimized errors that can occur during the ceramic restoration, there is still no clarity on whether silane-containing universal adhesives are as effective as silane treatment [4,7,12]. Therefore, the purpose of this study is to investigate the effect of silane-containing universal adhesives on the bonding strength of lithium disilicate. The null hypothesis of this study is as follows. Application of silane after using a silane-containing universal adhesive will not significantly affect the bonding strength of lithium disilicate.

## 2. Materials and Methods

### 2.1. Specimen Preparation

Table 1 summarizes the universal adhesives used in the experiment. Lithium disilicate with a diameter of 12 mm and height of 4 mm (LiSi Press; GC, Tokyo, Japan) was prepared and a silicone mold of the same size was made. The mold was filled with 2-mm-thick layers of composite resin (Charmfil flow; Dentkist, gyeonggi-do Korea). Each layer was light-cured for 20 s using a 1200 mW/cm^2^ LED light curing unit (Bluephase G2; Ivoclar Vivadent, Inc., Amherst, NY, USA) to produce a resin disc.

### 2.2. Etching, Silanization, and Bonding Procedure

**Figure 1:**
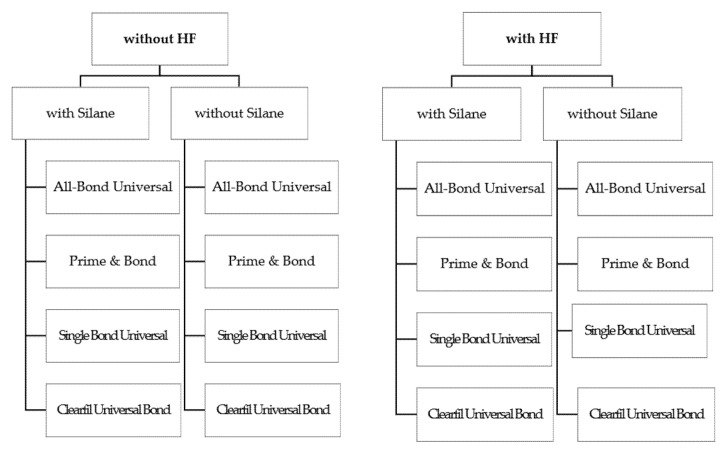
Group descriptions.

**Figure 2:**
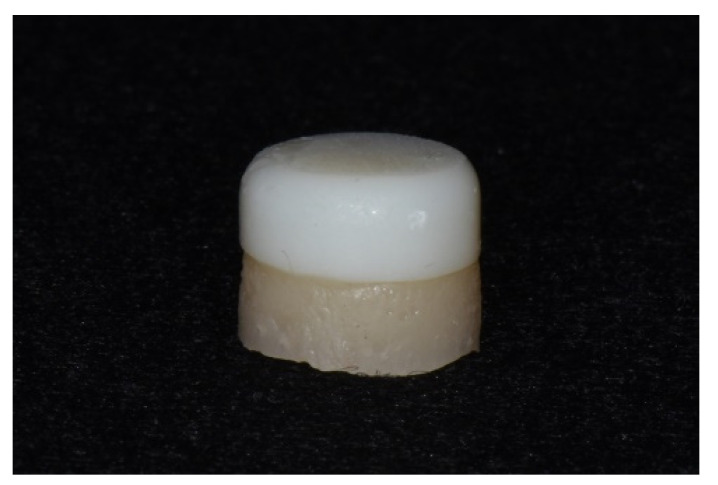
Specimens used in the experiment (Top: LiSi Press, Bottom: Charmfil flow).

Before cementation of lithium disilicate and the resin disc, specimens were divided into 16 groups (*n* = 15), depending on the type of universal adhesive used (ABU, PB, CUB, or SBU) and whether the specimens were treated with HF and/or silane (Figure 1). The group consisting of specimens untreated with any universal adhesives was set as a control group. A dried ceramic surface was treated with 9.5% HF (PORCELAIN ETCHANT; Bisco, Schaumburg, IL, USA) for 90 s and washed with water. The HF-treated surface was then treated once with silane (Porcelain Primer; Bisco, Schaumburg, IL, USA) for 30 s and dried using a three-way syringe. A universal adhesive was then applied following the manufacturer’s instructions. Subsequently, a dual-curing composite resin cement (RelyX™ Ultimate; 3M ESPE, St Paul, MN, USA) was applied to bonding the lithium disilicate ceramic with the resin disc. The resin disc was placed on the lithium disilicate and a weight of 2 g was used to keep the units in position during the removal of excess cement using a microbrush. The specimen was light-cured for 40 s using a LED light-curing unit (BluePhase G2; Ivoclar Vivadent Inc., Amherst, NY, USA) to obtain cemented ceramic with resin disc (Figure 2). Specimens stored in distilled water (37 °C) at room temperature for 24 h.

### 2.3. Microtensile Bond Strength (μTBS) Test

**Figure 3:**
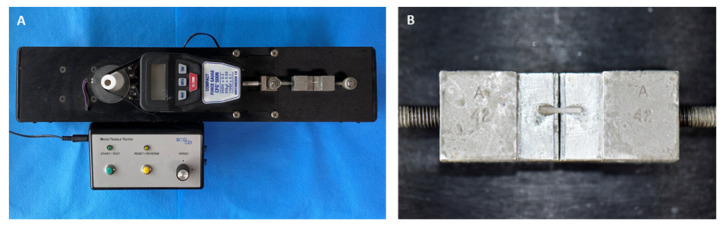
(**A**) µTBS tester. (**B**) Specimen is mounted on the specimen holder.

Specimens were sectioned into a 1 mm^2^ cross-sectional stick using a low-speed diamond saw (Accutom-50; Struers, Rødovre, Denmark). After the specimens were dried, both ends of the specimens were fixed on the jig of a µTBS tester (Micro Tensile tester; Bisco, Schaumburg, IL, USA) (Figure 3). A microtensile force was applied at a crosshead speed of 1.0 mm/min, and µTBS (MPa) was measured.

### 2.4. Analysis of Failure Mode

The fractured surface of all specimens was examined at 80× magnification under a stereomicroscope (Global A6 Series; Global surgical corporation, St. Louis, MO, USA). For failure modes, “adhesive failure” was defined as a failure between the ceramic and resin cement, “cohesive failure” as a failure within the ceramic or a resin disc, and “mixed failure” as a simultaneous observation of both adhesive and cohesive failures.

### 2.5. Microscopic Observation of Bonded Interfaces

**Figure 4:**
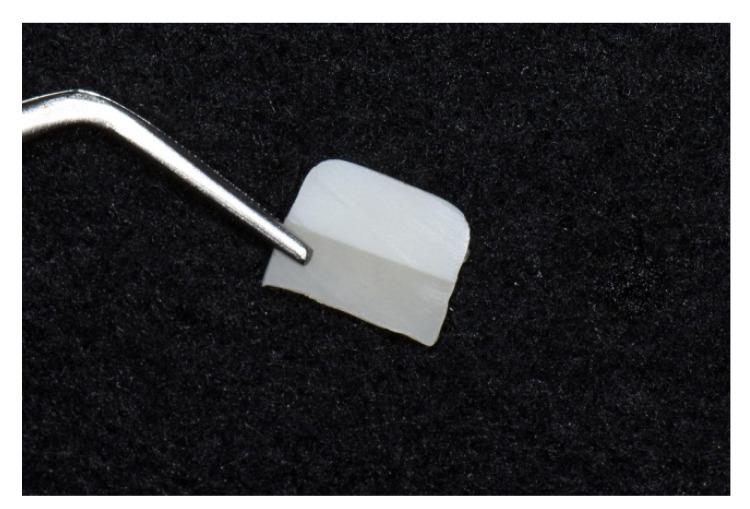
Sectioned ceramic–resin block (Top: LiSi Press, Bottom: Charmfil flow).

To examine the ceramic–resin block interface, ceramic–resin blocks were prepared via the aforementioned pretreatment process. The blocks were stored in distilled water at room temperature for 24 h and sectioned in the direction of the major axis using a low-speed diamond saw to prepare 8 × 8 × 1 mm^3^ specimens (Figure 4). The middle area of specimen from the sectioned block was used to examine the interface between the ceramic and the resin disc under a field-emission scanning electron microscope (FE-SEM) (JSM-7200F; JEOL Ltd., Tokyo, Japan).

### 2.6. Statistical Analysis

Statistical analyses were performed using SPSS 21.0 software (SPSS Inc., Chicago, IL, USA). One-way ANOVA and the Tukey test were used to compare μTBS between the groups, and the Student’s *t*-test was used to compare μTBS within the groups. (α = 0.05)

## 3. Results

### 3.1. *μ*TBS

**Figure 5:**
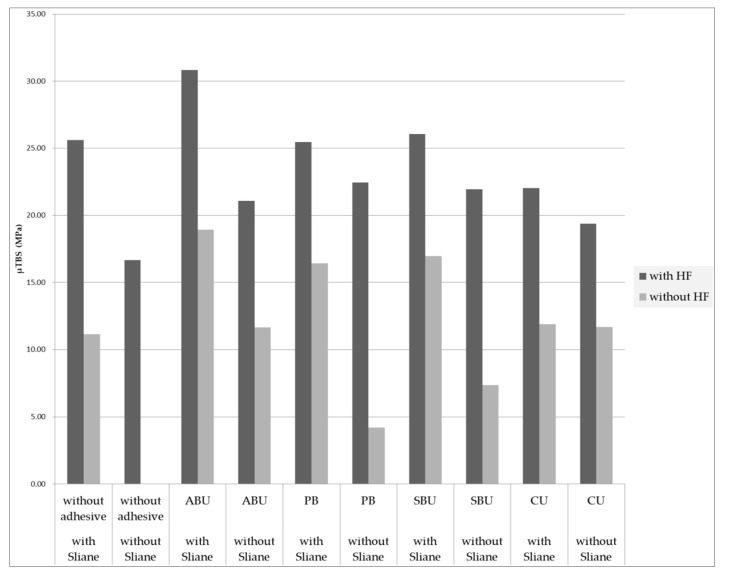
Graph of μTBS (MPa) according to HF application (*n* = 15).

Figure 5 and Table 2 present the μTBS results of each group. All groups treated with HF showed higher bonding strengths compared to those that were not treated with HF, regardless of silane treatment (*p* < 0.05). While the control, ABU, and SBU groups treated with HF showed increased bonding strengths after silane application (*p* < 0.05), the PB and CU groups showed no significant difference in the bonding strength following silane application (*p* > 0.05). All groups untreated with HF showed a significant increase in the bonding strength after silane application, except the CU group (*p* < 0.05).

### 3.2. Analysis of Failure Mode

Table 3 presents the distribution of failure modes. Adhesive failures were predominant across all groups. Some specimens in the ABU group treated with HF and silane showed mixed failures.

### 3.3. FE-SEM Evaluation

**Figure 6:**
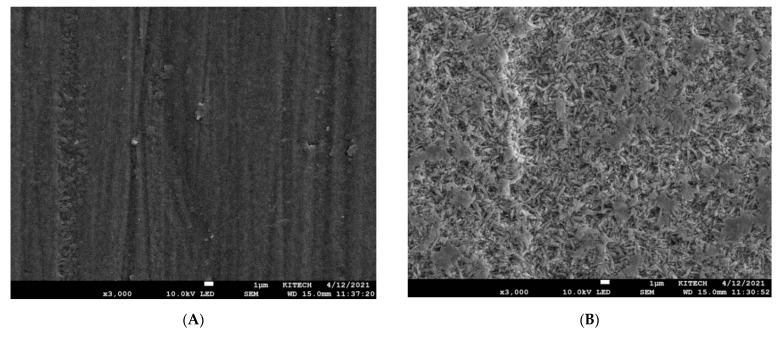
SEM images (×3000 magnification) of the LiSi press surface before (**A**) and after (**B**) treatment with 9.5% HF.

Figure 6 presents the FE-SEM images of lithium disilicate surfaces before and after HF treatment. Unetched specimens showed smooth and uniform surfaces without retentive irregularities (Figure 6A). On the other hand, the specimens treated with 9.5% HF showed clear changes in their surface morphologies due to the removal of the glass matrix from the surface of lithium disilicate and the exposure of lithium disilicate crystals (Figure 6B).

**Figure 7:**
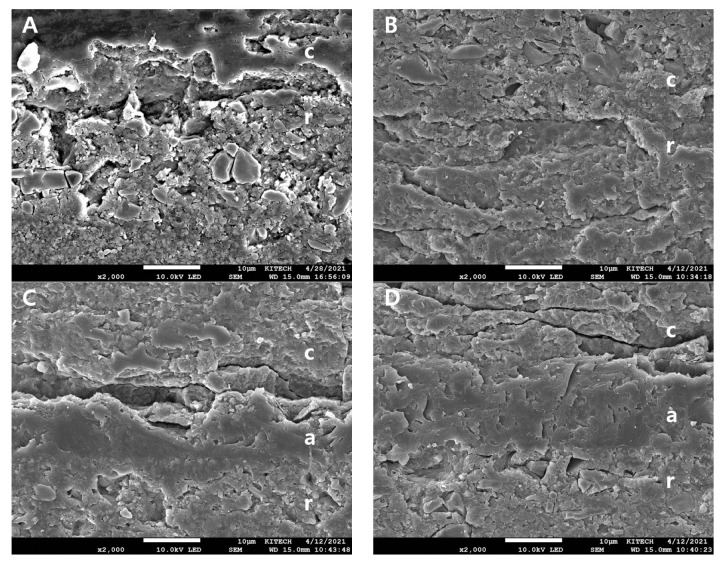

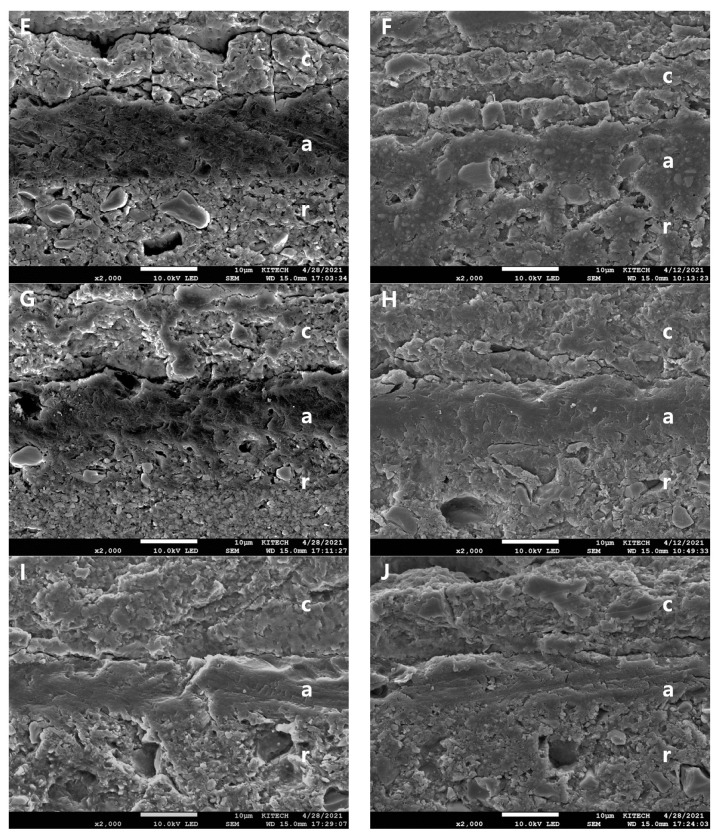
Comparison of the SEM images (×2000 magnification) of the interfaces between ceramic and resin cement in the specimens. (**A**) HF only, (**B**) HF and silane, (**C**) HF and ABU, (**D**) HF, silane, and ABU, (**E**) HF and PB, (**F**) HF, silane, and PB, (**G**) HF and SBU, (**H**) HF, silane, and SBU, (**I**) HF and CU, (**J**) HF, silane, and CU. c = ceramic, a = adhesive, r = resin cement.

Figure 7 presents the microscopic observations of the interfaces between lithium disilicate and resin discs. The specimens that were not treated with silane after HF treatment showed loose bonding between the ceramic and resin cement due to partial gaps (Figure 7A). On the other hand, the specimens treated with silane after HF application showed an infiltration of the resin cement into the micro-undercut of the ceramic surface, thereby creating a tight ceramic–resin interface (Figure 7B). The ABU and SBU groups treated with silane after HF application (Figure 7D,H) showed a deeper infiltration of the resin cement into the ceramic surface, as compared to that observed for the groups treated with HF but not with silane (Figure 7C,G). The PB and CU groups showed no differences in the bonding interface according to silane treatment (Figure 7E,F,I,J).

## 4. Discussion

The increasing interest in all-ceramic restorations and advances in bonding techniques have led to the development of universal adhesives containing a bifunctional agent called silane. Several studies have raised concerns about the instability that occurs during storage of saline-containing universal adhesives and the potential adverse effect it may have on bonding strength [12,13,14]. Therefore, the present in vitro study was conducted to investigate the actual effect of silane in universal adhesives as well as the effect of an additional silane application.

In this study, HF treatment improved the bonding strength between the resin and lithium disilicate, regardless of silane application; this has also been observed in previous studies [7,15,16,17,18,19]. HF (5–10%) is commonly used to treat the inner surface of a ceramic restoration. HF partially dissolves the ceramic glass matrix to expose the crystalline structure and induce mechanical interlocking between the ceramic and resin cement. Among the control groups, which were untreated with adhesives, the group treated with HF only (16.67 ± 5.18 MPa) showed a higher bonding strength compared to the one treated with silane only (11.15 ± 2.27 MPa); however, the group treated with HF and silane showed an even higher bonding strength (25.61 ± 4.60 MPa). Without silane treatment, the bonding strength of lithium disilicate depended on the micromechanical retention created by HF etching. An additional silane treatment strengthened the chemical bond between lithium disilicate and resin cement, thereby increasing their bonding strength. Based on these results of this study, the etching patterns on a ceramic surface created by HF and the resulting micromechanical retention are more important than the chemical bonding induced by silane, with regard to increasing the bonding strength between the ceramic and resin. Therefore, HF pretreatment may be essential to improve the bonding potential of ceramic restorations.

In general, silane with a bifunctional structure is applied after the glassy matrix is partially removed from the surface of lithium disilicate using HF. The alkali group on silane that reacts with silica, which is a hyaline, exists in the inactive form (-OR) and becomes activated upon hydrolysis (SiOR → SiOH), thereby forming a network structure wherein ceramics and resin are three-dimensionally interlocked [1]. A water-free silane primer must be hydrolyzed and activated by mixing it with a hydrophilic acetic acid solution or an acidic adhesive.

Manufacturers of silane-containing universal adhesives claim that these adhesives can achieve sufficient chemical bonding when used with ceramics, without the need for ceramic primers. However, previous study has reported that water-containing or acidic pre-hydrolyzed silane coupling agents have relatively short storage period [1], and the water-contact angle of silane increases with a decrease in the bonding strength when silane and methacrylate monomers exist within a single solution [12]. It has also been reported that MDP, which is a monomer commonly contained in universal adhesives and has a pH of 2–2.7, promotes self-condensation of silanol groups in the presence of silane and neutralizes the silane [11].

A key finding of this study is that silane-containing universal adhesives do not achieve a high bonding strength compared to that achieved using silane separately. In addition, by using non-silane-containing universal adhesives and silane together, a higher bonding strength is achieved than that obtained using universal adhesives alone; this is possibly because acidic MDP contained in universal adhesives neutralizes silane and makes it unstable over time [11]. Thus, it is thought that silane contained in universal adhesives is practically ineffective. Another possible explanation for this finding is that bisphenol A diglycidylmethacrylate (bis-GMA), which is another monomer present in universal adhesives, interferes with the condensation between the silanol groups of silane and the OH groups of ceramics [12]. Additionally, universal adhesives may not contain sufficient amounts of silane, which is one of their many constituents [5]. Thus, it can be concluded that using silane primers independently is a more effective after HF treatment rather than silane-containing universal adhesives.

In this study, when silane was applied separately, the bonding strength of all universal adhesive groups was improved, except for CU. In particular, an additional silane treatment after the application of a silane-containing universal adhesive (SBU or CU) significantly improved the bonding strength in the SBU group. Similar results were reported in a previous study, in which additional silane application yielded better µSBS values than silane-containing universal adhesive application alone [10]; however, this was not observed in the CU group. In this study, CU showed relatively low bonding strengths compared to those of SBU, which is consistent with previous reports [4]. The difference in the bonding strengths between the two adhesives may be attributed to the lack of clarity of their compositions, ratios, and concentrations. SBU contains polyalkenoic acid copolymers (Vitrebond copolymer; 3M ESPE) and has been reported to improve bonding strengths [4]. Colloidal silica is added in CU as a filler to achieve adequate resin thickness and viscosity; this addition reduces the flow of the adhesive layer, causes the adhesive to be partially concentrated in some areas and creates voids, and may be responsible for the reduced bonding strength and effectiveness of silane in the CU group [20]. Thus, the hypothesis that an additional silane application will not affect the effectiveness of silane-containing universal adhesives can be rejected.

In this study, all universal adhesives improved the bonding strength between the ceramics and resin cement, regardless of silane application. Previous studies have reported that a thin and unfilled resin layer increases the infiltration of resin cement into the irregular surface of a ceramic, thereby increasing the bonding strength between lithium disilicate glass ceramics and resin cement and improving interfacial quality [21]. The universal adhesives used in this study may have improved interfacial bonding by filling the voids in the ceramic surface, which cannot be filled with resin cement due to its relatively high viscosity. This led to an even surface and an evenly distributed stress across the surface.

The silane-containing universal adhesives did not achieve higher bonding strengths compared to those of the non-silane-containing universal adhesives, and this was in line with previous research [22]. ABU, which is a non-silane-containing universal adhesive, showed an even interface without a gap between the ceramics and resin cement in FE-SEM observation. This high-quality bonding demonstrates that ABU exhibits the highest bonding strength among all adhesive groups. Based on these results, it can be established that the bonding strength of a universal adhesive is more dependent on the product than on the presence of silane. Thus, determining whether a product contains silane may not be that important when choosing a universal adhesive for ceramic cementation.

The main limitation of this in vitro study was measuring the bonding strength between ceramic and resin, not teeth. This was to prevent pre-failure that may occur at the tooth–resin cement interface. Therefore, more studies similar to the actual clinical situation for longer time periods are needed in the future.

In conclusion, silane must be applied after HF treatment to ensure a high bonding durability between the lithium disilicate ceramics and resin cement. It has also been established that silane-containing universal adhesives cannot replace silane. Furthermore, it is not advisable to use additional universal adhesives after the application of HF and silane from an efficiency point of view, as doing so will not significantly affect bonding strengths except for ABU.

## 5. Conclusions

Within the limits of this study, the following conclusions can be drawn.

1. HF improved the bonding strength between lithium disilicate and resin regardless of the silane treatment.
2. For all the experimental groups that were treated with silane separately, the bonding strength between lithium disilicate and resin was improved.
3. The silane in universal adhesives did not effectively improve the bonding strength between lithium disilicate and resin cement, and the silane-containing universal adhesives were not more effective than the non-silane-containing universal adhesives in improving the bonding strength between lithium disilicate and resin cement.

## Figures and Tables

**Table 1:** Universal adhesives used in the experiment and their application methods.

Universal Adhesive	Abbreviation	Composition	Application Method	pH
All-Bond Universal (Bisco)	ABU	MDP, Bis-GMA, HEMA, ethanol, water, initiators	Apply for 10–15 s, mild air-dry for at least 10 s, light cure for 10 s	3.2
Prime & bond (DENTSPLY Caulk)	PB	MDP, Bis-GMA, HEMA, CQ, MCAP, D3MA, ethanol, water, highly dispersed silicon dioxide	Apply for 20 s, mild air-dry for 5 s, light cure for 10 s	2.5
Single Bond Universal (3M)	SBU	MDP, HEMA, silane, filler, dimethacrylate resins, water, Vitrebond copolymer, ethanol, initiators	Apply adhesive for 20 s, dry gently for 5 s, light cure for 10 s	2.7
Clearfil Universal Bond (Kuraray)	CUB	MDP, Bis-GMA, HEMA, CQ, hydrophilic aliphatic imethacrylate, colloidal silica, accelerators, silane coupling agent	Apply for 10 s, mild air-dry for 5 s, light cure for 10 s	2.3

MDP: 10-methacryloyloxydecyl dihydrogen phosphate, Bis-GMA: 2,2-Bis [4-(2-hydroxy-3-methacryloxypropoxy) phenyl] propane, HEMA: 2-hydroxyethyl methacrylate, CQ: camphorquinone, MCAP: Methacrylated carboxylic acid polymer, D3MA: Decandiol dimethacrylate.

**Table 2:** μTBS values (MPa) in each group (*n* = 15).

**With HF**	**ABU**	**PB**	**SBU**	**CU**	**Without Adhesive**
with Sliane	30.82 ± 4.72 ^b^	25.47 ± 2.75 ^a^	26.06 ± 3.86 ^ab^	22.03 ± 4.48 ^a^	25.61 ± 4.60 ^a^
without Sliane	21.07 ± 3.34 ^a^	22.45 ± 6.04 ^a^	21.95 ± 3.58 ^a^	19.39 ± 4.02 ^a^	16.67 ± 5.18 ^a^
*p*-value	*p* < 0.05	*p* > 0.05	*p* < 0.05	*p* > 0.05	*p* < 0.05
**Without HF**	**ABU**	**PB**	**SBU**	**CU**	**Without Adhesive**
with Sliane	18.92 ± 5.04 ^b^	16.44 ± 2.79 ^b^	16.97 ± 2.77 ^b^	11.89 ± 2.62 ^a^	11.15 ± 2.27 ^a^
without Sliane	11.65 ± 3.03 ^d^	4.21 ± 1.42 ^b^	7.37 ± 3.36 ^c^	11.68 ± 2.41 ^d^	pre-failure ^a^
*p*-value	*p* < 0.05	*p* < 0.05	*p* < 0.05	*p* > 0.05	*p* < 0.05

Different superscript letters (^a,b,c,d^) indicate statistically differences within the same row (*p* < 0.05).

**Table 3:** Failure mode distribution.

			Mixed Failure	Adhesive Failure
with HF	with Sliane	without adhesive	0	15
	without Sliane		0	15
	with Sliane	ABU	4	11
	without Sliane		0	15
	with Sliane	PB	0	15
	without Sliane		0	15
	with Sliane	SBU	0	15
	without Sliane		0	15
	with Sliane	CU	0	15
	without Sliane		0	15
without HF	with Sliane	without adhesive	pre-failure	
	without Sliane		0	15
	with Sliane	ABU	0	15
	without Sliane		0	15
	with Sliane	PB	0	15
	without Sliane		0	15
	with Sliane	SBU	0	15
	without Sliane		0	15
	with Sliane	CU	0	15
	without Sliane		0	15

## Data Availability

Data sharing not applicable.
